# IL-1*β* Impaired Diabetic Wound Healing by Regulating MMP-2 and MMP-9 through the p38 Pathway

**DOI:** 10.1155/2021/6645766

**Published:** 2021-05-18

**Authors:** Jiezhi Dai, Junjie Shen, Yimin Chai, Hua Chen

**Affiliations:** Department of Orthopedic Surgery, Shanghai Jiao Tong University Affiliated Sixth People's Hospital, Shanghai, China

## Abstract

Diabetes mellitus is one of the most prominent metabolic disorders in the world, and insulin resistance in diabetic patients leads to several complications including increased inflammation and delayed wound healing. Fibroblast migration and reepithelialization play a significant role in wound healing. In this study, we explored the effects of IL-1*β* signaling on proliferation and migration of human fibroblasts from diabetic wound tissues. We observed elevated levels of IL-1*β* in samples from diabetic patients when compared to normal wound tissues. At high concentrations, IL-1*β* inhibited cell proliferation and migration in *ex vivo* fibroblast cultures. Moreover, expression of matrix metalloproteinases (MMPs) was upregulated, and tissue inhibitor of metalloproteinases (TIMPs) was downregulated in diabetic wound tissues and cells. These effects were regulated by levels of IL-1*β*. Furthermore, IL-1*β* induced p38 phosphorylation thereby activating the p38 MAPK pathway that in turn regulated the expression of MMPs and TIMPs. Together, our study identifies a novel mechanism behind delayed wound closure in diabetes mellitus that involves IL-1*β*-dependent regulation of cell proliferation and migration.

## 1. Introduction

Diabetes mellitus is one of the most prevalent chronic diseases worldwide [1, 2]. In China, 11% of adults lived with diabetes in 2010 that amount to a total of 109.6 million individuals [3]. With a rapid increase in the prevalence of diabetes mellitus, complications such as diabetic ulcers have become major causes of deformity worldwide [4]. Of the different symptoms associated with diabetes, impaired wound healing is one of the most prominent effects observed in patients. Wound healing is an evolutionarily conserved process that involves spatial and temporal overlap of processes such as inflammation, coagulation, cellular proliferation, and extracellular matrix (ECM) remodeling [5–8].

Matrix metalloproteinases (MMPs) are ECM remodeling proteins that play a significant role in various stages of the wound healing process. A regulated metalloproteinase activation and inhibition cascade is triggered following injury for efficient wound closure [9]. Initially, MMPs are involved in the removal of devitalized tissue following which during the repair phase, MMPs contribute to angiogenesis, contraction of the wound matrix, migration of fibroblasts and keratinocytes, and epithelialization. Finally, they are also involved in the remodeling of newly synthesized connective tissue [9, 10]. MMP activity is regulated by small inhibitory proteins known as tissue inhibitor of metalloproteinases (TIMPs), which also contribute to the initiation of cell division and binding to the ECM as well as the inhibition of angiogenesis and the induction of apoptosis [11]. Hence, the balance between MMP and TIMP activity is crucial for effective wound healing [12, 13].

Serum levels of IL-1*β* are elevated in patients with diabetes [14]. Previous studies identified the contribution of IL-1*β* towards persistent inflammation in chronic wounds and its role in impaired wound healing [15]. However, the effects of IL-1*β* signaling in the context of diabetic wound healing have not been studied so far. Therefore, in this study, we explored the role of IL-1*β* in diabetic wound closure using patient tissues and *ex vivo* fibroblast cultures and identified a novel mechanism behind delayed wound healing observed in patients with diabetes mellitus.

## 2. Methods

### 2.1. Clinical Specimens

All patients were recruited from the Department of Orthopedic Surgery, Shanghai Jiao Tong University Affiliated Sixth People's Hospital. Patients characterized for type 2 diabetes with chronic wounds located on the foot and nondiabetic patients (control group) with a leg wound were enrolled in our study. Patients presenting chronic wounds resulting from pressure ulcer, pyoderma gangrenosum, vasculitis, and other diseases that cause ischemia were excluded. Serum and wound tissue samples of nondiabetic (*n* = 30) and type 2 diabetic (*n* = 30) patients were collected. This study was approved by the Ethic Review Board of Shanghai Six People's Hospital affiliated to Shanghai Jiao Tong University (YS-2019-010) and was in accordance with the principles of the declaration of Helsinki as amended. Written informed consent was obtained from each participant.

### 2.2. Cell Culture

To culture fibroblasts *ex vivo*, human tissue specimens were cut into 1-3 mm^3^ cubes, digested in 0.15% collagenase (Roche Applied Science, Indianapolis, IN, USA), and cultured in DMEM medium (Gibco, Gaithersburg, MD, USA) at 37°C for 3 h [16]. Single-cell suspensions were then filtered through nylon meshes and centrifuged at 1500 rpm for 5 min. Pellet containing fibroblasts was resuspended and seeded in 10 cm plates in fully supplemented DMEM containing 10% fetal bovine serum (Invitrogen, Carlsbad, CA, USA). Cells were cultured at 37°C and 5% CO_2_. Confluent cells were passaged and subcultured at 1 : 3 ratio. Cells were harvested for analysis after the first passage.

### 2.3. Mouse Skin Wound Samples

Male C57BL/6J mice were purchased from the Model Animal Research Center of Nanjing University. Diabetic (db/db) mice were created by knocking out the gene coding for the leptin receptor. The mice were maintained under 12 h light/12 h dark cycles and have unrestricted access to food and water at a controlled temperature (25°C). The skin wound samples were collected as previously described. Briefly, 8-week-old mice were anesthetized using ether following which the hair of the back skin was removed with a hair clipper. Two circular full-thickness wounds were made on the skin with a 6 mm skin biopsy trephine (Kai Industries, Inc., Gifu, Japan). On day 7 postwounding, mice were euthanized by cervical dislocation and 2 mm peripheral skin from the wounded region was collected. The skin samples were immediately immersed in liquid nitrogen and stored at ˗80°C until further use. All animal experiments have been approved by the Animal Care and Use Committee of the Shanghai Jiao Tong University and guidelines on animal care and use according to the National Institute of Health (NIH, USA) guidelines were followed.

### 2.4. Quantitative Real-Time PCR

Total RNA from cells or tissues was extracted using TRIzol and was subsequently reverse transcribed by RevertAid First Strand cDNA Synthesis Kit. Quantitative real-time PCR was performed using SYBR Green PCR mix on an ABI Prism 7500HT (Applied Biosystems) with GAPDH as negative control. The primer pairs used were as follows: hIL-1*β*, ATGATGGCTTATTACAGTGGCAA and GTCGGAGATTCGTAGCTGGA; hMMP2, TACAGGATCATTGGCTACACACC and GGTCACATCGCTCCAGACT; hMMP9, GGGACGCAGACATCGTCATC and TCGTCATCGTCGAAATGGGC; hTIMP1, AGAGTGTCTGCGGATACTTCC and CCAACAGTGTAGGTCTTGGTG; hTIMP2, AAGCGGTCAGTGAGAAGGAAG and GGGGCCGTGTAGATAAACTCTAT; hGAPDH, AGCCACATCGCTCAGACAC and GCCCAATACGACCAAATCC; mIL-1*β*, GCAACTGTTCCTGAACTCAACT and ATCTTTTGGGGTCCGTCAACT; mMMP2, CAAGTTCCCCGGCGATGTC and TTCTGGTCAAGGTCACCTGTC; mMMP9, CTGGACAGCCAGACACTAAAG and CTCGCGGCAAGTCTTCAGAG; mTIMP1, GCAACTCGGACCTGGTCATAA and CGGCCCGTGATGAGAAACT; mTIMP2, TCAGAGCCAAAGCAGTGAGC and GCCGTGTAGATAAACTCGATGTC; and mGAPDH, AGGTCGGTGTGAACGGATTTG and TGTAGACCATGTAGTTGAGGTCA.

### 2.5. Western Blotting

Tissues and cells were lysed with lysis buffer containing 50 mM Tris-HCl (pH 6.8), 2% sodium dodecyl sulfate, 10 mM dithiothreitol, 10% glycerol, 0.002% bromophenol blue, and protease inhibitor cocktail (Roche, Basel, Switzerland). Total protein quantification was performed using Pierce BCA Protein Assay Kit (ThermoFisher Scientific). Equal amounts of protein were loaded and separated by sodium dodecylsulfate (SDS)-polyacrylamide gel electrophoresis and transferred onto PVDF membranes (Millipore, Billerica, MA, USA). Membranes were blocked in 5% bovine serum albumin or nonfat milk and incubated with antibodies overnight at 4°C. The primary antibodies against IL-1*β*, MMP2, MMP9, TIMP1, TIMP2, collagenase I, collagenase III, p-p38 mitogen-activated protein kinase (MAPK), p-protein kinase B (AKT), AKT, p-phosphatidylinositol 3 kinase (PI3K), PI3K, and *β*-actin were purchased from Cell Signaling Technology (Danvers, MA, USA). After 3x washing with TBS-Tween 20, the membranes were incubated with secondary antibodies coupled to horseradish peroxidase for 1 hour at room temperature. Protein bands were visualized using chemiluminescence.

### 2.6. Cell Viability Assay

CCK8 assay was performed to analyze cell viability. Cells were seeded in 96-well plates in quintuplicates at a density of 1 × 104 cells/well and maintained in a humidified atmosphere of 5% CO_2_ at 37°C. The plated cells were incubated with IL-1*β* at 5, 50, or 100 ng/mL concentration for different times. Cells were then treated with 10 *μ*L/well CCK8 solution (Dojindo Molecular Technologies, Japan) for 2 hours. The OD450 in each well was determined by a microplate reader, reflecting the amount of total viable cells under each condition.

### 2.7. Migration Assay

A scrape wound assay was used to evaluate cell motility. Normal healthy fibroblasts or fibroblasts were seeded from diabetic patients in 6 cm dishes. Confluent cells were scraped using a sterilized 200 *μ*L tip, and nonadherent cells were washed off with medium. The healthy fibroblast cells were then treated with the indicated dose of IL-1*β* for 72 hours. Cells in the migrated fraction was photographed using a microscope, and the percentage of total area covered by the cells in each image was calculated using ImageJ (National Institutes of Health, USA). Three independent experiments were performed.

### 2.8. ELISA

Measurement of IL-1*β* in the serum from clinical samples or mice was performed using human or mouse IL-1*β* Quantikine ELISA Kit (R&D systems), respectively, according to manufacturer's instructions.

### 2.9. Statistical Analysis

Data was represented as mean ± standard deviation (SD) from at least three or more independent experiments. Statistical significance was calculated using Student's *t*-test or one-way ANOVA followed by Tukey's post hoc test. *p* ≤ 0.05 was considered as statistically significant.

## 3. Results

### 3.1. IL-1*β* Levels Were Increased in the Wounds of Patients with Diabetes

**Figure 1:**
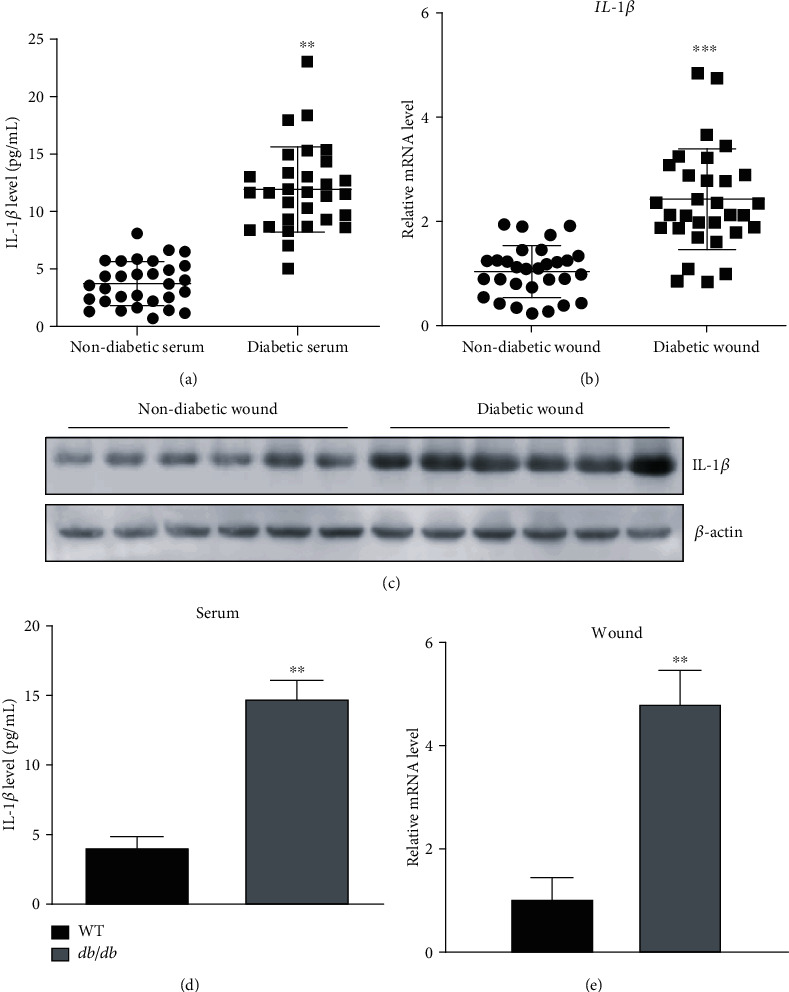
IL-1*β* levels were increased in diabetic wound tissues: (a) levels of IL-1*β* in the serum of patients as detected by ELISA; (b) mRNA levels of IL-1*β* in wound tissues as measured by qPCR; (c) protein levels of IL-1*β* in wound tissues; (d, e) IL-1*β* levels in serum and wound tissues in WT and db/db mice. ^∗∗^*p* < 0.01; ^∗∗∗^*p* < 0.001.

To study the role of IL-1*β* in wound healing in patients with diabetes, we collected serum and wound tissues from normal and diabetic individuals and performed ELISA/western blots to measure protein levels of IL-1*β* in the serum and tissues, respectively, and qPCR to measure IL-1*β* mRNA levels. We found that IL-1*β* was upregulated in the serum of diabetic patients (Figure 1(a)). Consistently, mRNA and protein levels were also increased in wound tissues of diabetic patients when compared to normal tissues (Figures 1(b) and 1(c)). We then evaluated levels of IL-1*β* in wound tissues from db/db type 2 diabetic wound healing mouse model and observed that expression of IL-1*β* was significantly higher in the serum and wounds from diabetic mice when compared to normal mice (Figures 1(d) and 1(e)). Together, these results indicate that IL-1*β* is upregulated in wound tissues under diabetic conditions.

### 3.2. High Levels of IL-1*β* Inhibit Proliferation and Migration of Fibroblasts

**Figure 2:**
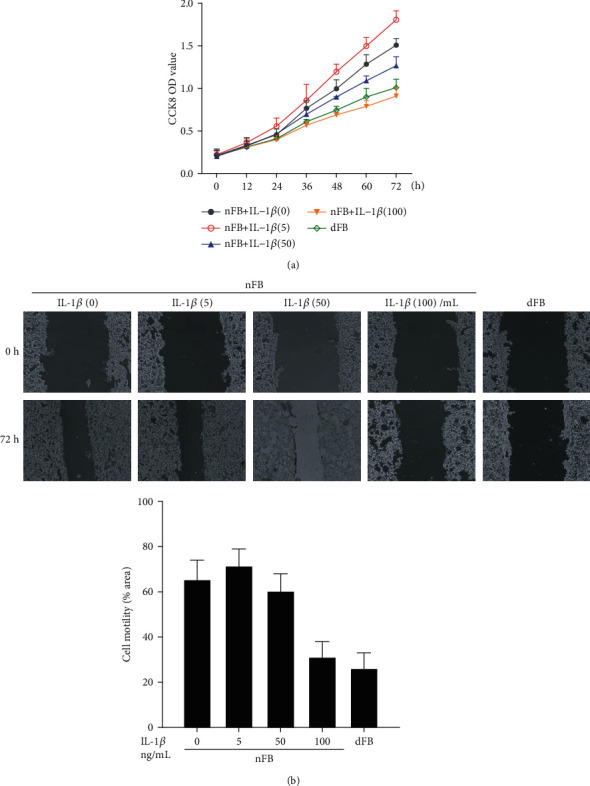
High dose of IL-1*β* inhibits proliferation and migration of fibroblasts *ex vivo*: (a) CCK8 assay indicating cell viability of normal (nFB) and diabetic fibroblasts (dFB) under different concentrations of IL-1*β* (0, 5, 50, 100 ng/mL); (b) cell proliferation of fibroblasts under different concentrations of IL-1*β*. Representative images and quantification are shown. ^∗^*p* < 0.05.

We cultured primary fibroblasts from normal or diabetic wound tissues *ex vivo* and observed a decrease in cell proliferation in diabetic fibroblasts (dFB) when compared to normal fibroblasts (nFB) (Figure 2(a)). We then evaluated the effect of IL-1*β* on fibroblast proliferation and observed that low dose of IL-1*β* had no effect or promoted cell proliferation whereas a higher dose significantly inhibited proliferation of fibroblast (Figure 2(a)). Further, scrape wound assay was used to determined cell motility and we found that dFB displayed impaired migration capability when compared to nFB. The effect on migration was dependent on levels of IL-1*β* as we observed a decrease in cell migration with increasing concentrations of IL-1*β* (Figure 2(b)). Taken together, these results indicate that high levels of IL-1*β* impair cell proliferation and migration in fibroblasts *ex vivo*.

### 3.3. Expression of MMPs Is Upregulated in Diabetic Wounds whereas TIMP Proteins Are Downregulated

**Figure 3:**
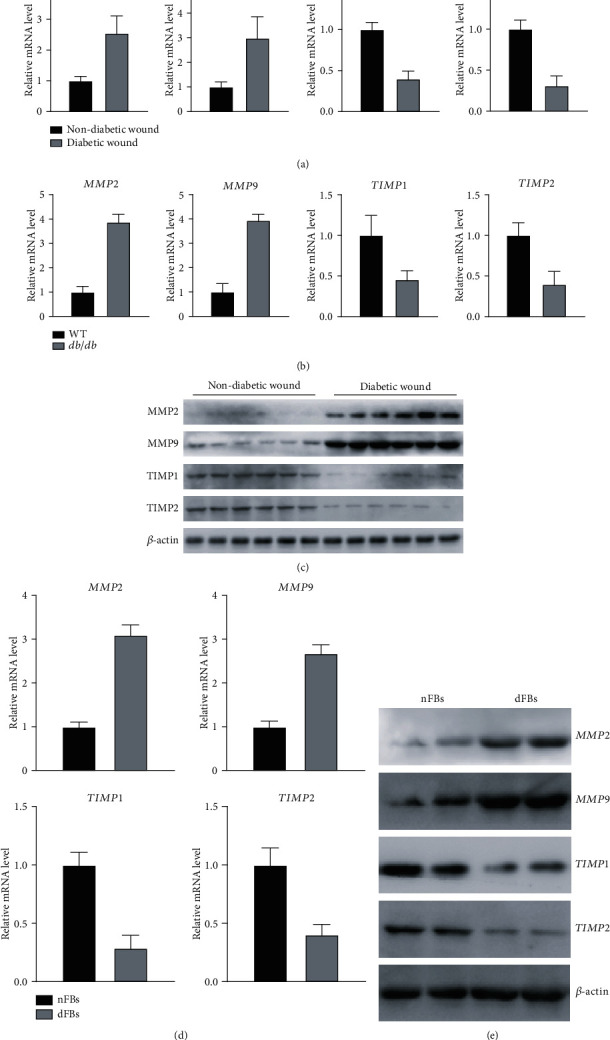
Diabetic wounds display elevated MMP expression and reduced TIMP expression: (a) mRNA levels of MMP2, MMP9, TIMP1, and TIMP2 in nondiabetic and diabetic wound tissues from patients evaluated by qPCR; (b) mRNA levels of MMP2, MMP9, TIMP1, and TIMP2 in control and db/db wound tissues from mice; (c) protein expression of MMP2, MMP9, TIMP1, and TIMP2 in nondiabetic and diabetic wounds evaluated by western blot; (d, e) mRNA quantification (d) and protein expression (e) in nFB and dFB samples. ^∗^*p* < 0.05.

As several studies showed that MMPs were potential predictive markers for impaired wound closure in diabetic foot ulcers [17], we next measured protein and mRNA levels of MMP-2, MMP-9, TIMP1, and TIMP2 in tissues and cultured fibroblasts from diabetic wound patients and compared them with the normal control group. We found that the mRNA expression of MMP2 and MMP9 was significantly upregulated in the wounds of diabetic patients while the expression of TIMP1 and TIMP2 that are inhibitors of MMP2 and MMP9, respectively, was significantly downregulated in diabetic wounds when compared to normal wounds (Figure 3(a)). We then evaluated TIMP1 and TIMP2 expression in wound tissues of db/db and control mice and observed a similar effect (Figure 3(b)). These results were further confirmed at the protein level by western blot using samples from the wounds of diabetic and nondiabetic patients. MMP2 and MMP9 protein expression was significantly higher in diabetic wound samples when compared to nondiabetic wounds, while TIMP1 and TIMP2 expression levels were significantly lower in diabetic wound tissues (Figure 3(c)). We then validated these results in cultured fibroblasts and observed that both mRNA and protein levels of MMP2 and MMP9 were upregulated in dFBs while TIMP1 and TIMP2 expression was downregulated (Figures 3(d) and 3(e)). Taken together, our results indicate that diabetic conditions increase expression of MMP proteins in wound tissues while simultaneously downregulating expression of TIMP proteins.

### 3.4. IL-1*β* Induced Expression of MMPs and Downregulated Expression of TIMP Proteins *Ex Vivo*

**Figure 4:**
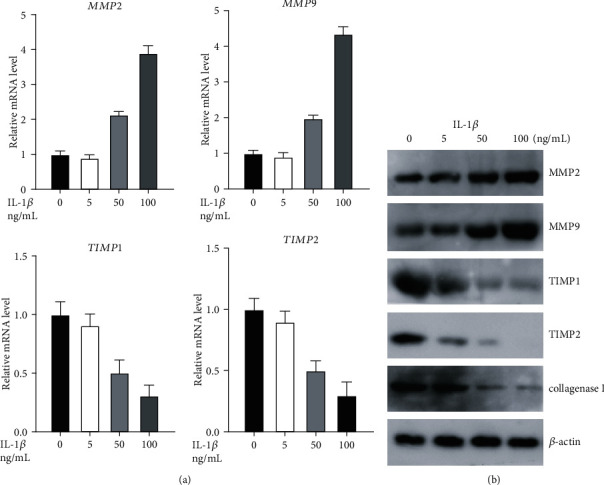
IL-1*β* induces MMP expression and inhibits TIMP expression *ex vivo*: (a, b) mRNA and protein levels of MMP2, MMP9, TIMP1, and TIMP2 in nFB at various doses of IL-1*β*. ^∗^*p* < 0.05.

We next evaluated the effects of IL-1*β* on the expression of MMPs and TIMPs in primary fibroblasts *ex vivo*. IL-1*β* was found to upregulate the expression of MMP2 and MMP9 in a dose-dependent manner as quantified by qPCR, while simultaneously inhibiting the expression of TIMP1 and TIMP2 (Figure 4(a)). We observed similar results when measuring protein expression by western blot. IL-1*β* inhibited the expression of collagenase I which facilitates wound healing along with TIMP1 and TIMP2 while simultaneously promoting the expression of MMP2 and MMP9 (Figure 4(b)). These results indicate that levels of MMP and TIMP proteins that are involved in the process of wound healing are directly regulated by IL-1*β*.

### 3.5. IL-1*β* Expression Regulates the Activation of p38 MAPK Pathway

**Figure 5:**
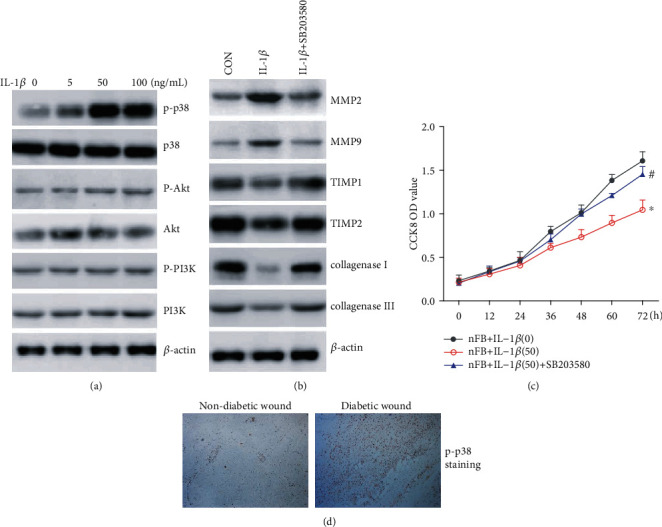
IL-1*β* activates the p38 MAPK pathway to mediate its effects in wound healing. (a) Activation of p38 MAPK, PI3K, and AKT was measured upon IL-1*β* treatment. (b) p38 MAPK inhibitor SB203580 (10 *μ*M) was used to treat nFB. Protein levels of MMP2, MMP9, TIMP1, and TIMP2 were measured. (c) p38 MAPK inhibitor SB203580 (10 *μ*M) was used to treat nFB. Cell proliferation of fibroblasts was detected. (d) p-p38 MAPK staining in diabetic and nondiabetic wounds.

We measured the effects of IL-1*β* treatment on the activation of p38 MAPK, PI3K, and AKT pathways in cultured fibroblasts. IL-1*β* treatment increased phosphorylation of p38 in a dose-dependent manner but not AKT and PI3K (Figure 5(a)). We next measured the expression levels of MMPs and TIMPs in the presence and absence of p38 MAPK inhibitor SB203580 (10 *μ*M) and observed that treatment with an inhibitor reduced the effects of IL-1*β* on the expression of ECM remodeling proteins MMPs and TIMPs and collagenase (Figure 5(b)). Furthermore, inhibitor treatment also reduced the effect of IL-1*β* on cell proliferation (Figure 5(c)). Phosphorylated p38 expression was significantly higher in sections from diabetic wounds when compared to nondiabetic wound samples (Figure 5(d)). Together, these results indicate that IL-1*β* regulates the expression of ECM remodeling proteins in wound tissues via the p38 MAPK pathway in diabetes mellitus.

## 4. Discussion

In the present study, we confirmed the increase in levels of IL-1*β* in patients with type 2 diabetes mellitus that was also previously reported by several studies [18, 19]. Current theories regarding the pathophysiology of diabetes conceptualize the disease as a proinflammatory state characterized, among other things, by elevated levels of IL-1*β*. Blockade of the effects of IL-1*β* is therefore a promising focus of study in diabetes therapeutics [20]. Toll-like receptors and the Nod-like receptor protein-3 (NLRP3) inflammasome, as well as interleukin-1*β*, all appear to participate in the pathogenesis of diabetes [21]. Several studies have highlighted the importance of IL-1 in the pathogenesis of type 2 diabetes and identified it as a promising therapeutic strategy [22–25]. Although Mandrup-Poulsen and the AIDA study group (2013) reported that IL-1 inhibitor treatment is not effective against type 1 diabetes [26], its outcomes in mitigating type 2 diabetes remain to be evaluated. In order to pursue this approach as an efficient treatment strategy, the molecular mechanisms underlying the effects of IL-1 in type 2 diabetes is necessary.

The role of IL-1*β* in diabetic wound healing has been described previously. Mirza et al. reported that IL-1*β* plays a key role in sustaining the proinflammatory macrophage phenotype and in impairing the healing of diabetic wounds [15]. Treatment with an inhibitor of IL-1 receptor reversed the impaired wound healing process in a diabetic mouse model [27]; however, the mechanism behind this effect was not elucidated. We observed that elevated levels of IL-1*β* in diabetic wound tissues triggers the secretion of MMPs and inhibits expression of TIMP proteins to interfere with ECM remodeling and wound closure. In the process of pulmonary fibrosis, IL-1*β* stimulates the proliferation of fibroblasts and the production of type I and III collagen [28]. Furuyama et al. described that IL-1*β* induces the secretion of MMP-2 and MMP-9 in fibroblasts, thereby inhibiting the formation of basement membrane [29], which is in accordance with our results. These findings indicate that IL-1*β* regulates the formation and degradation of ECM in fibroblasts; however, the discrepancy between the studies may be due to the levels of IL-1*β* used as we also observed an inhibitory effect on fibroblast proliferation and migration only under high concentrations, as observed in diabetic patient samples, whereas low concentrations of IL-1*β* promoted cell proliferation.

Remodeling of the ECM is a primary requirement for wound closure [30] and MMPs are an important family of cell migration-related proteins that can degrade different components of the ECM [31]. Our results showed that both mRNA and protein levels of MMPs were higher in diabetic wounds than those in the control group, while levels of TIMP were lower. These results suggest that diabetic wounds are characterized by greater breakdown of ECM, a phenomenon that delays wound healing. It is proven by many studies that levels of MMPs are higher in the exudates of chronic wounds than in those of acute wounds, as in diabetic foot ulcers [32]. Addition of IL-1*β* stimulated the expression of MMPs and inhibited the production of TIMPs and collagenase suggesting that IL-1*β* promotes ECM degradation and delays the process of wound healing in diabetes.

We also observed that the action of IL-1*β* may at least in part be mediated by the p38 MAPK pathway. It is well known that p38 MAPK is capable of regulating a variety of cellular responses to cytokines and stress, including IL-1*β* [33]. p38 was found to promote the invasion and migration of gastric adenocarcinoma cells by increasing the levels of MMPs [34]. The addition of IL-1*β* to cultured fibroblasts resulted in increased phosphorylation of p38 in a dose-dependent manner whereas it showed no effect in AKT and PI3K. Phosphorylated p38, the activated form of p38, was also significantly higher in sections from diabetic wound tissues than in nondiabetic wounds and treatment with p38 MAPK inhibitor SB203580 reversed the effects of IL-1*β* on ECM remodeling protein expression.

## 5. Conclusions

Taken together, our study suggests that IL-1*β* mediates delayed wound healing in diabetic patients by altering levels of ECM remodeling proteins through activation of the p38 MAPK pathway thereby impairing cell proliferation and migration. It also identifies IL-1*β* as a potential therapeutic target to treat delayed wound closure in type 2 diabetic patients.

## Data Availability

The data used to support the findings of this study are included within the article, which are available from the corresponding author upon request.

## Abbreviations

MMP: Matrix metalloproteinase
TIMP: Tissue inhibitor of metalloproteinase
NLRP3: Nod-like receptor protein-3
ECM: Extracellular matrix
MAPK: Mitogen-activated protein kinase
AKT: Protein kinase B
PI3K: Phosphatidylinositol 3 kinase.
